# Synthesis and characterization of hydroxyapatite nanoparticles and their effects on remineralization of demineralized enamel in the presence of Er,Cr: YSGG laser irradiation

**DOI:** 10.1186/s12903-023-03549-z

**Published:** 2023-10-30

**Authors:** Fatemeh Maddah, Mehdi Shirinzad, Zahra Khalafi, Loghman Rezaei-Soufi, Younes Mohammadi, Fatemeh Eskandarloo, Abbas Farmany

**Affiliations:** 1 Department of Restorative Dentistry, School of Dentistry, Hamadan University of Medical Sciences, Hamadan, Iran; 2 Department of Biostatistics, School of Public Health and Research Center for Health Sciences, Hamadan University of Medical Sciences, Hamadan, Iran; 3 Dental Research Center, Research Institute of Dental Sciences, Shahid Beheshti University of Medical Sciences, Tehran, Iran; 4 Dental Research Center, School of Dentistry, Hamadan University of Medical Sciences, Hamadan, Iran; 5 Dental Implant Research Center, School of Dentistry, Hamadan University of Medical Sciences, Hamadan, Iran

**Keywords:** Hydroxyapatite nanoparticles, ER, CR: YSGG laser, Remineralization, Demineralized enamel

## Abstract

**Background:**

This study aims to synthesize and characterize hydroxyapatite nanoparticles (nano-HA) and evaluate their effects on the remineralization of demineralized enamel in the presence to Er,CR: YSGG laser irradiation.

**Materials and methods:**

Enamel specimens from 44 human molars were divided into four groups: control, demineralized enamel, demineralized enamel treated with nano-HA, and demineralized enamel treated with nano-HA followed by Er,Cr:YSGG laser irradiation (0.5, 20 Hz, 60 µs, 20 s). Vickers microhardness test was used to evaluate the enamel surface hardness. The morphology and chemistry of enamel surfaces were assessed using scanning electron microscopy (SEM) and Raman spectroscopy, respectively.

**Result:**

The result of this study showed that the application of Er,CR: YSGG laser irradiation to demineralized enamel treated with nano-HA had the highest impact on its microhardness.

**Conclusion:**

ER,CR: YSGG laser irradiation promotes enamel remineralization after treatment with nano HA.

## Introduction

The main goals of dental healthcare are the prevention of dental caries and the arrest of carious lesions. In the oral cavity, the tooth structure is subjected to demineralization and remineralization processes, and any disruption to the chemical balance in the oral environment can damage the tooth structure [1, 2].

Reversing early enamel defects is an important aspect of prevention, as it involves a dynamic process that alternates between demineralization and remineralization, ultimately resulting in the apparent repair of the lesion [3]. Currently, some methods have been designed to mimic the natural remineralization process for treating enamel defects. In preventive dentistry, nanotechnology is used as an antibacterial nanotherapy and biomimetic remineralization to reverse incipient caries or recurrent decay. Composite systems containing ACP, HA, TTCPs, mono-, di-, and tricalcium phosphates are some of the methods currently available that mimic the remineralization process [4, 5].

Most of the evidence regarding therapeutic remineralization involves the use of fluoride [6]. However, fluoride alone has not been shown to be an effective remineralizing agent in preventing tooth decay. Furthermore, there is controversy surrounding whether this treatment improves the milky appearance of porous enamel or just re-hardens the surface layer with less effect on its appearance [7].

Hydroxyapatite (HA) is a biocompatible material that is widely used in medicine and dentistry [8]. In dentistry, this compound is used in surface treatment of implants and production of hydroxyapatite-containing cements [9]. Microcrystalline HA particles are suitable for the prevention of demineralization or remineralization of demineralized enamel and dentin [10].“

Hydroxyapatite (HA) is the primary component of enamel, providing a bright white appearance and closing small enamel surface pores to eliminate diffuse reflectivity of light. It is one of the most biocompatible and bioactive dental materials [11].

Biomimetic approaches have been employed in recent years to develop nanomaterials for remineralizing early enamel lesions [12]. Nano-HA, in comparison to HA, exhibits unique properties such as higher solubility, higher surface energy, and greater biocompatibility [13]. In addition, laser therapy has emerged as an adjunct method for preventing caries and treating initial defects by altering the enamel structure and chemistry, increasing the tooth’s resistance to acidic challenges [7].

Although there have been studies on the effects of laser irradiation and nano-HA on enamel remineralization, there is limited data on the combined effects of both treatments for demineralized enamel. Therefore, the present study aimed to examine the effect of HA nanoparticles and Er,Cr:YSGG laser on the remineralization of demineralized enamel.

## Materials and methods

The study received ethics approval from the Ethics Committee of Hamadan University of Medical Sciences (IR.UMSHA.REC.1398.1031), and all methods were conducted in accordance with relevant guidelines and regulations.

### Sample preparation

Forty-four non-carious human third molars were extracted within the past three months and stored in a 0.05% chloramine solution (Merck, Germany). Soft tissue and calculus were removed from the teeth using a sharp blade, and the teeth were examined using a stereo microscope (Olympus, Shinjuku, Tokyo, Japan) at a magnification of 40× for any cracks or lesions. The crowns were then sectioned at the cementoenamel junction (CEJ) using a diamond blade, and the facial and palatal halves were separated. Each half was mounted on acrylic resin (Achropars, Iran), ground flat, and hand-polished using aqueous slurries containing progressively finer grades of silicon carbide, up to 4,000 grit (Struers). A surface area of 4 × 4 mm was exposed, while the remaining area was protected with acid-resistant nail polish [14]. To avoid the dehydration, the samples were immersed in distilled water.

### pH cycling

The samples were kept in a demineralization solution (2.2 mmol NaH_2_PO_4_, 2.2 mmol CaCl_2_, 0.1 ppm NaF, and 50 mmol acetic acid; pH = 4.6) for 3 h and then immersed in the remineralization solution (20 mmol HEPES, 130 mmol KCl, 1.5 mmol CaCl_2_, 0.9 mmol KH_2_PO_4_, and 1 mmol NaNO_3_; pH = 7) for 21 h. This cycle was repeated for 14 days. The demineralization and remineralization solutions were renewed every two day [5].

### Study groups

All samples were randomly divided into four groups: Group 1 (control): the enamel was demineralized, and then the samples were immersed in distilled water. Group 2: samples were demineralized and treated with nano-HA (0.05) for 24 h. Group 3: samples were demineralized similarly to Group 1 and then immersed in nano-HA (0.05) for 24 h [14, 15]. Afterward, the samples were treated with Er,Cr:YSGG laser irradiation. Group 4: samples were demineralized and then treated with Er,Cr:YSGG laser irradiation. After remineralization, the samples were stored at 37 °C for seven days, and the surface of each sample was examined using SEM microscopy, Raman Spectroscopy, and microhardness.

### Synthesis of nano-hydroxyapatite

Hydroxyapatite nanoparticles (nano-HA) were synthesized using the sol-gel method [16]. Briefly, an appropriate amount of diammonium hydrogen phosphate and calcium nitrate were dissolved in deionized water, and after reaching a pH of 10.5, they were mixed dropwise. After forming a milky suspension, it was stirred for 3 h., washed with double-distilled water, and dried at 60 °C using an oven. The prepared sediment was calcined at 650 °C using an electric oven.

### Characterization of nano-hydroxyapatite

To examine the crystalline phase and structure of the synthesized nanoparticles, their X-Ray diffraction pattern (XRD) was obtained using a Panalytical Xpert PRO X Ray Diffractometer (Panalytical, Netherlands- Xpert Pro MPD) with a wavelength of 1.5405Å and power of 40KV/30mA. Fourier-Transform Infrared Spectroscopy (FTIR) was used to evaluate the chemical structure, chemical bond structure, and functional groups of synthesized nanoparticles. FTIR spectra of synthesized nano-HA were recorded using a Perkin Elmer Frontier FTIR (PerkinElmer, USA, Spectrum400) at 400–4000 cm^− 1^. The morphology and size of nanoparticles were examined using TEM (Philips XL30 ESEM-Netherlands).

### Laser radiation

Er,Cr:YSGG laser with a wavelength of 2780 nm, power of 0.5 W, pulse energy of 600 mJ, pulse width of 60 µs, frequency of 20 Hz, 60% water, 40% air, and an exposure time of 20 s using Gold Handpiece (MZ6- diameter of 600 μm and length of 4 mm) held perpendicular to the surface at a distance of 5 mm from the surface (non-contact) with a sweeping movement was used.

#### SEM micrography

To examine the morphology and surface structure of the samples, SEM was used. Before SEM imaging, the surface of each sample was covered with a thin layer of gold. The scan area included the cross-section of lesion and a part of the surface. The samples were examined with magnification of 20,000×.

### Microhardness measurement

The microhardness test was performed using Knoop diamond penetration (kg/m^2^). For this purpose, a 50 g load was applied for 10 s. Microhardness was measured at three points on the surface. The indentations were made on the tested enamel surface of the subsurface lesion side of each enamel block, and they were approximately 100 μm apart from each other. Then the mean microhardness was calculated for three points.

### Raman spectroscopy

To examine the chemistry of enamel surfaces, Raman spectroscopy was used. Raman spectra (Teksan, TakRam N1-541) were obtained for all the studied groups at three randomly selected points.

### Statistical analysis

For statistical analysis, the mean values and standard deviations were calculated, and the level of significance was set at p < 0.001. The one-way ANOVA was used to compare means between different groups, while the t-test was employed to determine significant differences between means of two unrelated groups. All statistical analyses were performed using SPSS version 17 (SPSS Inc., Chicago, IL, USA).

## Results

### Nanoparticles characterization

**Fig. 1:**
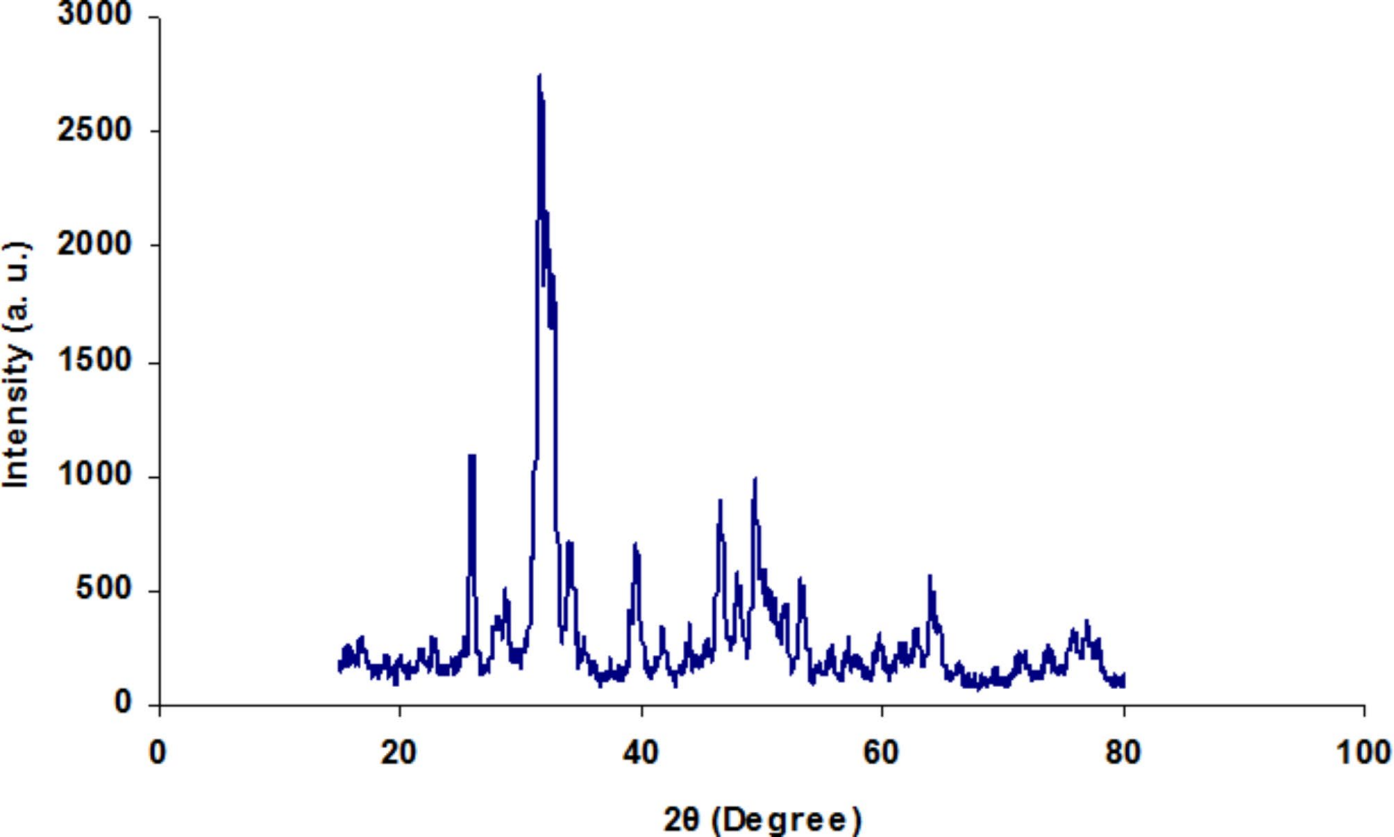
XRD pattern of nano-HA

The XRD profile of the synthesized nanoparticles is presented in Fig. 1. The pattern and position (2θ) of XRD peaks of calcified nano-HA at 650 °C clearly demonstrate its crystalline structure, consistent with JCPDS#896,438.

**Fig. 2:**
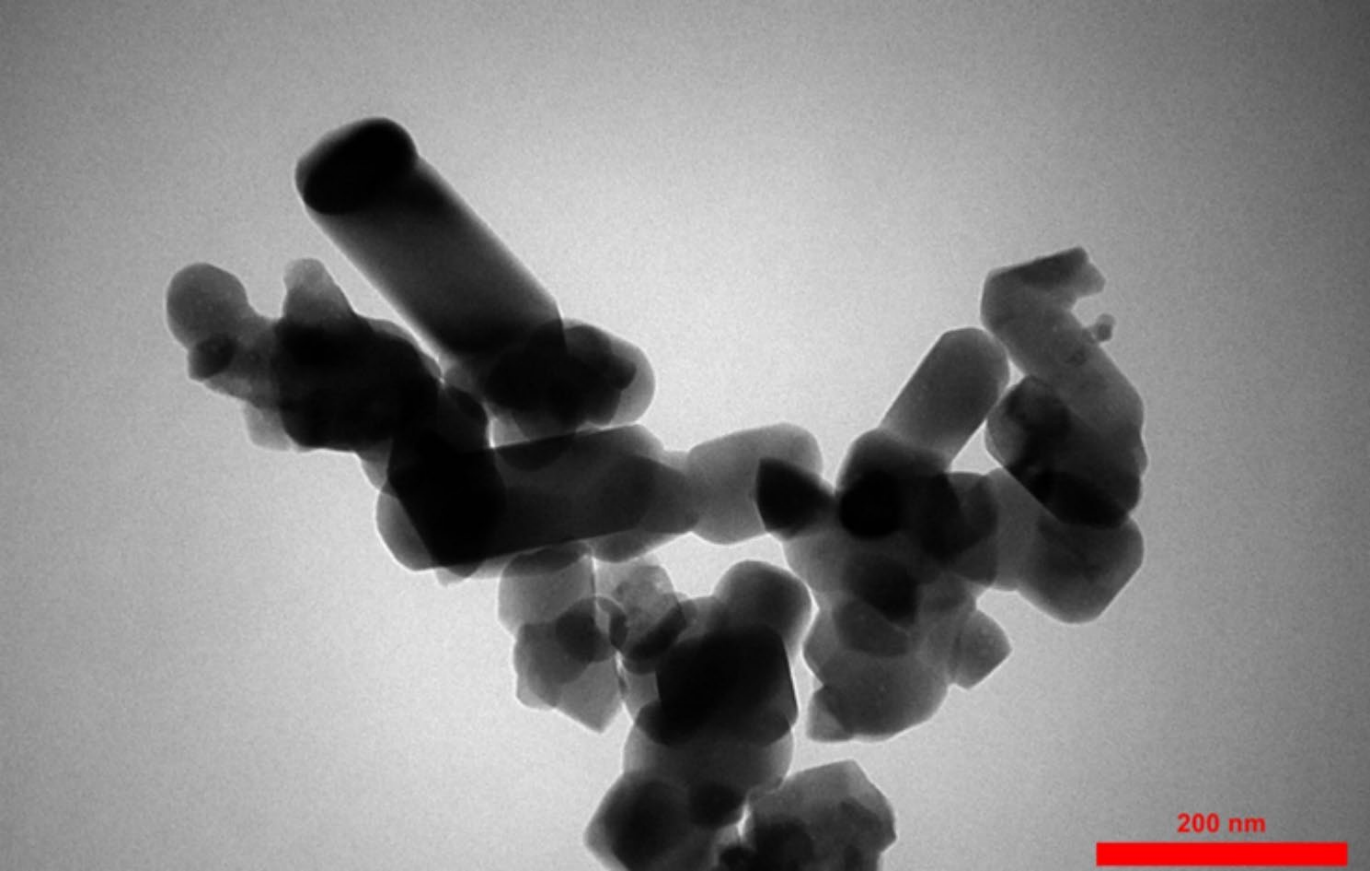
TEM microimage of nano-HA

To examine the structure and morphology of synthesized nano-HA, TEM was used. The TEM image of the synthesized nanostructures is shown in Fig. 2.

**Fig. 3:**
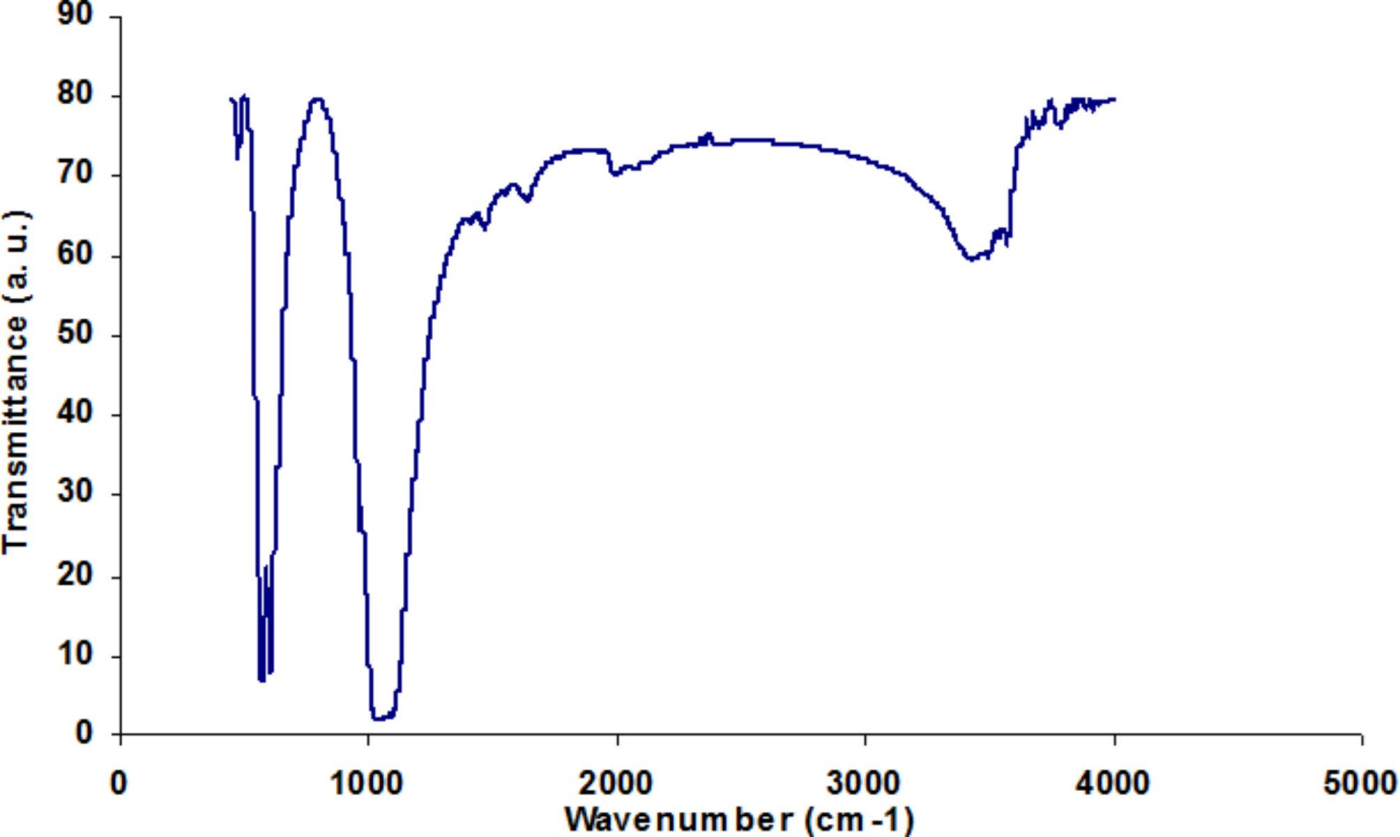
FTIR spectrum of nano-HA

FT-IR spectroscopy was used to confirm the formation and purity of the synthesized nanoparticles. Figure 3 illustrates the FTIR spectrum of the nano-HA sample. The PO_4_ ^− 3^ and CO_3_ ^− 2^ adsorption spectra of nano-HA are registered at 563, 1017, and 1445 cm^− 1^, respectively. The water content in the nano-HA sample is shown as hydroxyl adsorption at 3367 and 1612 cm^− 1^. Additionally, stretching vibration of -OH is observed at 3367 cm^− 1^.

### Microhardness analysis

**Fig. 4:**
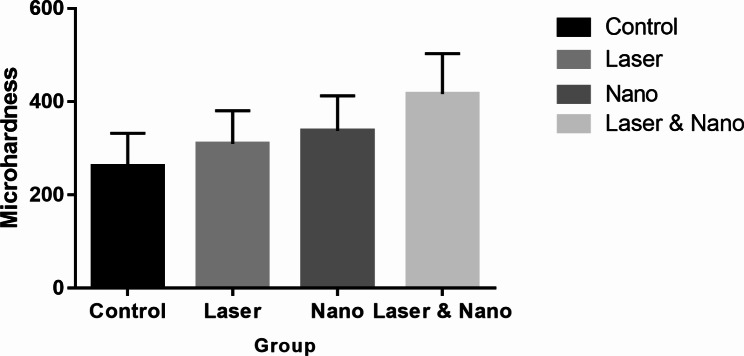
Microhardness of studied groups

The mean, standard deviation (SD), minimum, and maximum microhardness values of the studied groups are presented in Table 1. A one-way ANOVA test showed that the mean microhardness of the studied groups is significantly different (p = 0.001). *Post hoc* Tukey’s results are presented in Table 2. The results of this study show that the control group is not significantly different from the nano-HA and laser groups, while it is significantly different from the nano-HA + laser group. The nano-HA + laser group had a higher mean microhardness compared to the control and other groups (Fig. 4). Therefore, based on the microhardness data, exposure to laser irradiation after treatment with nano-HA had the highest impact on microhardness. Additionally, the lowest microhardness was observed in the control group.

**Table 1:** Mean, SD, min and max of microhardness

	N	Mean	Std. Deviation	Min	Max
Control	12	261.5833	70.86321	180.30	443.60
Laser	12	309.4250	70.88648	196.60	421.30
nano-HA	12	337.0000	75.23614	209.60	441.00
Laser & nano-HA	12	422.0000	86.80595	244.00	512.00
Total	48	331.0021	92.96607	180.30	512.00

**Table 2:** *Post hoc* Tukey results

group(I)	group(J)	Mean Difference (I-J)	Sig.	Confidence Interval (95%)	
				Lower Bound	Upper Bound
Control	Laser	47.8417	0.4245	-35.2477	130.9311
	nano-HA	75.4167	0.0874	-7.6727	158.5061
	Laser & nano-HA	160.4167	0.0000	77.3273	243.5061
Laser	nano-HA	27.5750	0.8120	-55.5144	110.6644
	Laser & nano-HA	112.5750	0.0041	29.4856	195.6644
Nano-HA	Laser & nano-HA	85.0000	0.0432	1.9106	168.0894

### SEM microimaging

**Fig. 5:**
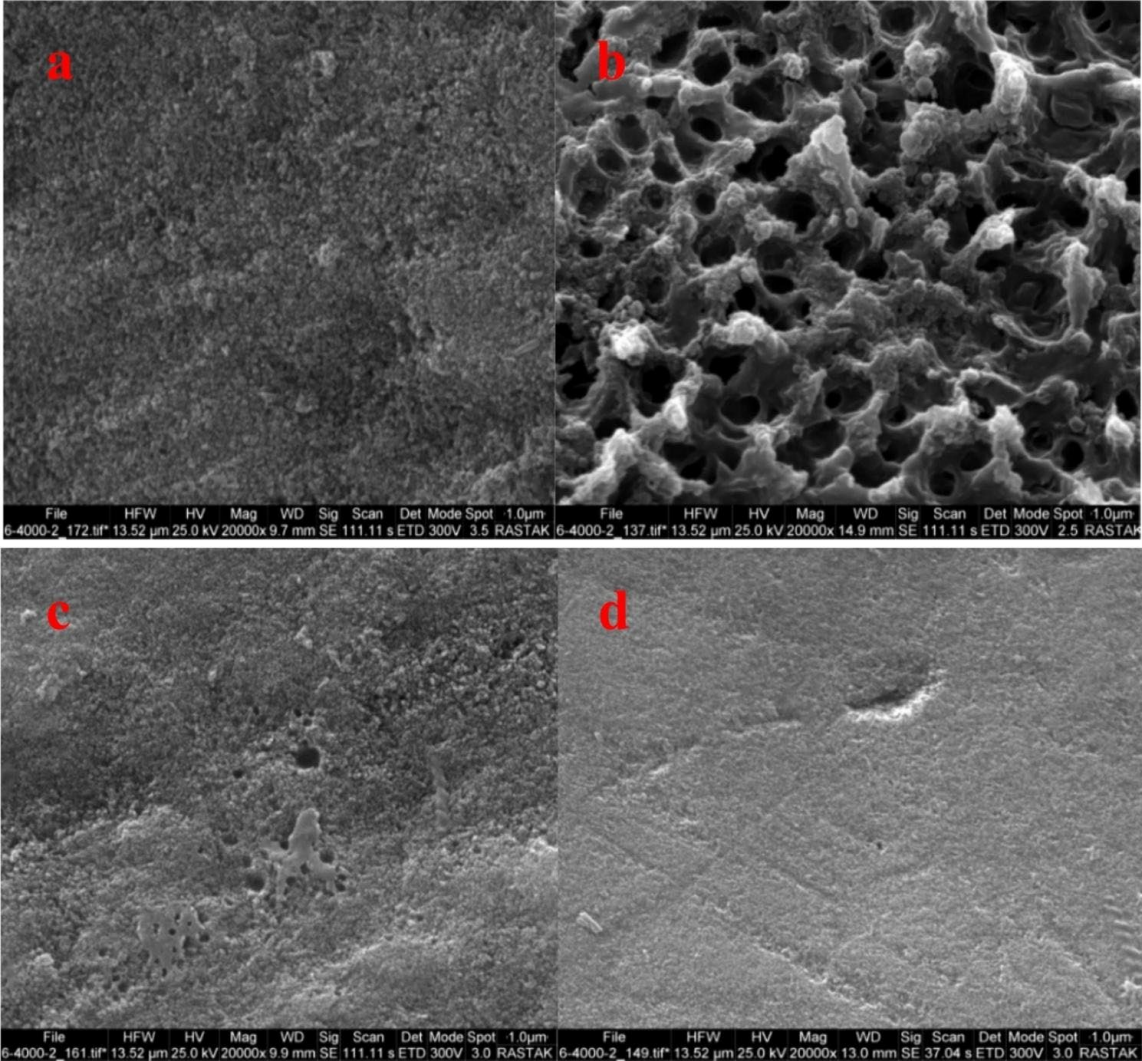
SEM microimage of: **(a)** Control group, **(b)** Laser group, **(c)** nano-HA group, and **d)** laser + nano-HA group

To examine the surface morphology of enamel, SEM micrographs of the tooth enamel surfaces were obtained (Fig. 5). As shown in Fig. 5a, honeycomb formation is visible on the surface of samples in the control group, which is due to enamel demineralization. In the laser group, the honeycomb formation is almost eliminated due to the melting and recrystallizing process of hydroxyapatites on the surface of enamel (Fig. 5b). Samples in the nano-HA group demonstrate a uniform sediment of minerals on enamel prisms, while the enamel surface is fully covered by a thin mineral layer (Fig. 5c). A similar phenomenon was observed in the nano-HA + laser group (Fig. 5d). Amorphous sedimentation of nano-HA is visible on the surface of enamel prisms, which the laser beam has melted and created a uniform distribution over the enamel. In this group, there is no honeycomb formation covering the enamel.

### Raman spectroscopy

**Fig. 6:**
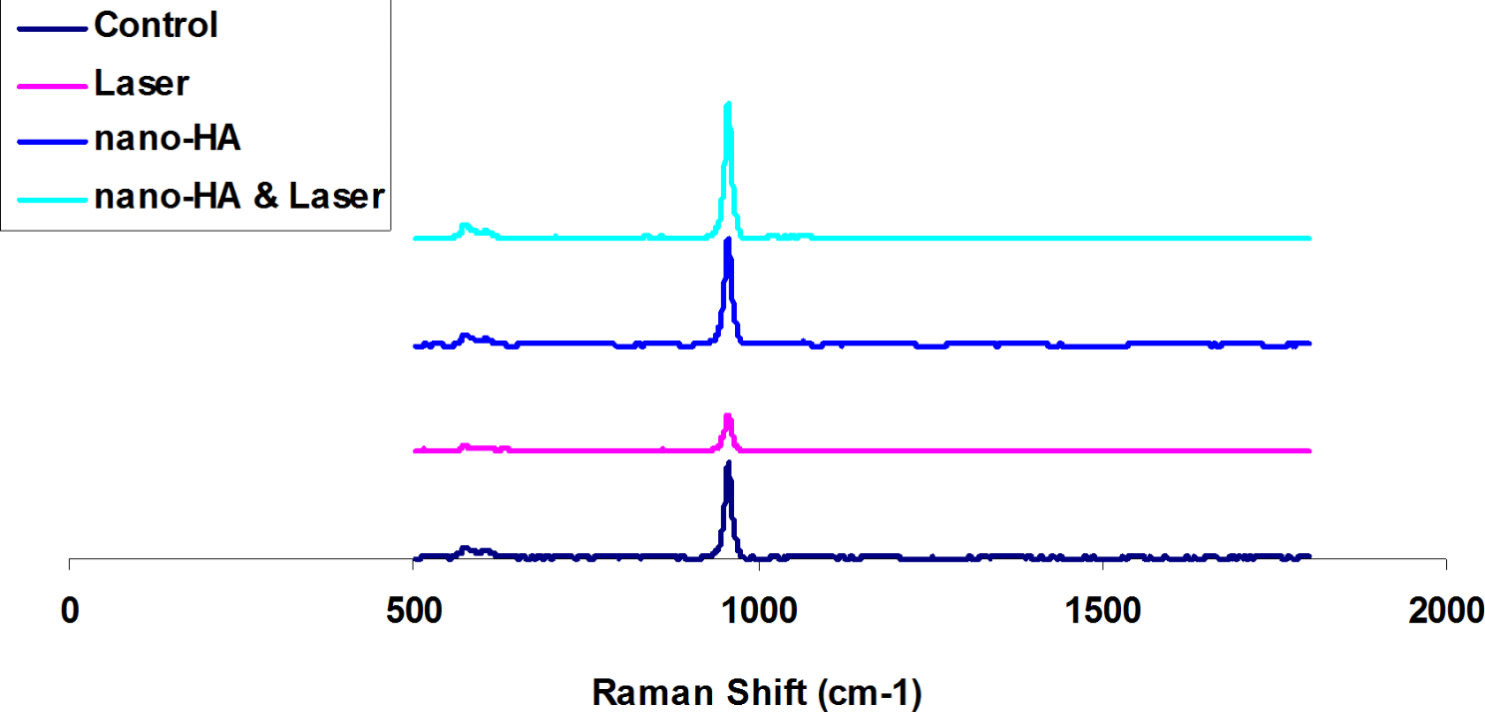
Raman spectra of studied groups

Raman spectrometry was used to quantitatively examine the chemistry and mineral profiles of the enamel surface. Figure 6 illustrates Raman spectra of enamel in studied groups at 500–1800 cm^− 1^. As shown in Fig. 6, a strong absorption peak is registered at 958 cm^− 1^ for all samples which refers to perfectly symmetrical stretch of phosphate ions (P-O). Because of the destruction of the enamel surface in the laser group, the absorption decreases notably, while the spectrum is stronger in the nano-HA, control, and nano-HA + laser groups. The obtained results indicate that using nano-HA in combination with laser improved remineralization process in the enamel surface.

## Discussion

In this study, microhardness assessment was used to evaluate the enamel surface remineralization as a sensitive measure of mineral loss. This technique is particularly suitable for surfaces like enamel with delicate, heterogeneous, and crack-prone microscopic structure. It is an easy, non-destructive, and fast method that can be used in demineralization and remineralization assessments [17].

Nano-HA induces enamel remineralization consistent with the formation of a homogeneous apatite layer on demineralized surfaces of enamel after treatment. This can be attributed to the hydrophilic and moisturizing properties of nano-HA, which enable it to produce a thin layer on the tooth surface, resulting in greater surface hardness and remineralization [18]. Moreover, nano-HA acts as a filler by repairing small holes and depressions on the enamel surface, a function that is enhanced by its nanometer size [11]. Min et al. demonstrated that the addition of nano-sized hydroxyapatite to a sports drink inhibits dental erosion in bovine enamel [19]. In our study, nano-HA improved enamel microhardness, although the difference between the nano-HA group and the control group was not statistically significant.

Kim et al. investigated the combined effects of nano-hydroxyapatite and NaF mouth rinses on the remineralization of early caries lesions. They found that the concentration of nano-HA positively correlated with the degree of remineralization [20]. These results support the remineralization ability of nano-HA, which is consistent with the findings of our study.

In vitro studies have demonstrated that high-potency (4–6 watts) Er,Cr:YSGG laser irradiation of hard tooth tissues significantly increases their acid resistance. Haghighi et al. investigated the effect of laser application on preventing demineralization during orthodontic treatment. They found that laser irradiation is a novel method for inhibiting demineralization around brackets and other orthodontic appliances, which can be combined with fluoride therapy [7].

The SEM study revealed that the affected areas had melted and appeared thermally degraded [21]. Under such circumstances, thermally degraded enamel showed little change after demineralization. Qiao et al. demonstrated the efficacy of Er,Cr:YSGG laser irradiation in increasing acid resistance of hard tooth tissue [22]. These studies suggest that high-energy radiation from lasers can cause surface melting of enamel, which, in turn, can facilitate fusion of nano-HA crystals on the enamel surface and inhibit enamel demineralization [7]. However, Kantorowitz and McCormack reported that melting of the enamel surface and fusion of nano-HA crystals might not be necessary to increase acid resistance and that high-energy laser irradiation could potentially raise the temperature above 1000 °C, which could be harmful to the pulp [5].

Another study by Apel et al. suggested that application of sub-ablative Er lasers may cause small cracks in enamel, which could act as a starting point for acid attack, deep demineralization, and ultimately, a reduction in the positive effect of enamel caries prevention. The discrepancies in the results of these studies may be attributed to differences in the laser settings used. Freitas et al. compared the effects of different power and fluencies of Er,Cr:YSGG laser and found that the best result in terms of enamel resistance to acid was achieved with 8.5 J/cm^2^, 0.75 W, and 20 Hz [23]. In our current study, the laser settings were similar to those used in the aforementioned study, and our results showed an improvement in enamel microhardness after laser application.

Our findings indicated a significant increase in enamel microhardness following exposure to nano-HA and laser irradiation. The increased microhardness of softened enamel after laser irradiation may be attributed to structural changes, including crystal size growth and recrystallization of porous enamel due to the high temperature rise on the surface [24]. It is possible that the chemical and structural alterations facilitate nano-HA deposition and improve the microhardness of the enamel surface [25].

Our study examined the effects of synthesized nano-HA and Er,Cr:YSGG laser on remineralization of primary enamel decay. The results demonstrated a significant increase in enamel microhardness following exposure to nano-HA and laser irradiation.

## Conclusion

In conclusion, our study demonstrated that the application of Er,Cr:YSGG laser after treatment of samples with nano-HA significantly increased the microhardness of the enamel surface. These findings suggest that the combination of nano-HA and laser therapy may hold promise as a potential approach to promote enamel remineralization and prevent dental caries. Further studies are warranted to explore the long-term effects and clinical applications of this approach.

## Data Availability

All supporting data are available upon request (Corresponding author).

## Abbreviations

HA: Hydroxyapatite
SEM: Scanning electron microscopy
XRD: X-Ray Diffraction
FTIR: Fourier-Transform Infrared spectroscopy
